# Isolation Rearing Reduces Neuronal Excitability in Dentate Gyrus Granule Cells of Adolescent C57BL/6J Mice: Role of GABAergic Tonic Currents and Neurosteroids

**DOI:** 10.3389/fncel.2016.00158

**Published:** 2016-06-13

**Authors:** Giuseppe Talani, Francesca Biggio, Valentina Licheri, Valentina Locci, Giovanni Biggio, Enrico Sanna

**Affiliations:** ^1^Institute of Neuroscience, National Research Council of ItalyMonserrato, Cagliari, Italy; ^2^Department of Life and Environmental Sciences, Section of Neuroscience and Anthropology, University of Cagliari, MonserratoItaly; ^3^Department of Biomedical Science, University of SassariSassari, Italy

**Keywords:** social isolation, GABAergic tonic current, glutamate, neuronal excitability, dentate gyrus

## Abstract

Early-life exposure to stress, by impacting on a brain still under development, is considered a critical factor for the increased vulnerability to psychiatric disorders and abuse of psychotropic substances during adulthood. As previously reported, rearing C57BL/6J weanling mice in social isolation (SI) from their peers for several weeks, a model of prolonged stress, is associated with a decreased plasma and brain levels of neuroactive steroids such as 3α,5α-THP, with a parallel up-regulation of extrasynaptic GABA_A_ receptors (GABA_A_R) in dentate gyrus (DG) granule cells compared to group-housed (GH) mice. In the present study, together with the SI-induced decrease in plasma concentration of both progesterone and 3α,5α-THP, and an increase in THIP-stimulated GABAergic tonic currents, patch-clamp analysis of DG granule cells revealed a significant decrease in membrane input resistance and action potential (AP) firing rate, in SI compared to GH mice, suggesting that SI exerts an inhibitory action on neuronal excitability of these neurons. Voltage-clamp recordings of glutamatergic spontaneous excitatory postsynaptic currents (sEPSCs) revealed a SI-associated decrease in frequency as well as a shift from paired-pulse (PP) depression to PP facilitation (PPF) of evoked EPSCs, indicative of a reduced probability of glutamate release. Daily administration of progesterone during isolation reverted the changes in plasma 3α,5α-THP as well as in GABAergic tonic currents and neuronal excitability caused by SI, but it had only a limited effect on the changes in the probability of presynaptic glutamate release. Overall, the results obtained in this work, together with those previously published, indicate that exposure of mice to SI during adolescence reduces neuronal excitability of DG granule cells, an effect that may be linked to the increased GABAergic tonic currents as a consequence of the sustained decrease in plasma and hippocampal levels of neurosteroids. All these changes may be consistent with cognitive deficits observed in animals exposed to such type of prolonged stress.

## Introduction

Prolonged stress induced by adverse events, including social isolation (SI), during early stages of life has been consistently associated with several deficits in the normal neurophysiological and neurodevelopmental processes in both humans and rodents (Hoffman-Plotkin and Twentyman, 1984; Feldman and Walton-Allen, 1997; Gottfried et al., 1998; McGue and Bouchard, 1998; Rutter, 1998; O’Connor et al., 2000; Kreppner et al., 2001). In rodents, deprivation of social interaction from weaning and during adolescence produces marked effects on brain development in several brain regions, including the hippocampus, that reach their complete developmental process in the second month of life (Lanier and Isaacson, 1977). For example, in the mouse dentate gyrus (DG), granule cells begin to proliferate at embryonic day 10 but most of this process occurs during the first 3 weeks of postnatal life (Angevine, 1965; Altman and Bayer, 1990) and still continue to be generated into adult life (Kempermann et al., 2015). Thus, exposure to stress during postnatal development may induce profound changes in the function of specific brain structures.

SI in rodents promotes an array of behavioral, neuroendocrine, and neurochemical changes (Hall et al., 1998; Ferdman et al., 2007; Pietropaolo et al., 2008) including a sustained decrease in plasma and brain concentrations of GABAergic neuroactive steroids such as allopregnanolone (3α,5α-THP) and 3α,5α-THDOC as well as an increased expression and function of extrasynaptic α4/δ-containing GABA_A_ receptors (GABA_A_R) in DG granule cells of both rats (Serra et al., 2000, 2005, 2006) and mice (Sanna et al., 2011). Neuroactive steroids produced by peripheral organs (Paul and Purdy, 1992), but also synthesized *de novo* in the brain (defined as neurosteroids; Hu et al., 1987; Mathur et al., 1993) from their precursor progesterone, regulate neuronal excitability acting mainly as potent positive modulators of GABA_A_R, particularly those containing the δ subunit that are located at extrasynaptic sites and are responsible for generating tonic inhibitory currents (Nusser et al., 1995; Glykys and Mody, 2007; Glykys et al., 2008).

Previous studies reported that SI may affect also neuronal excitability in the hippocampal formation (Bartesaghi and Serrai, 2001; Bartesaghi and Severi, 2002; Bartesaghi et al., 2003; Bartesaghi, 2004; Talani et al., 2011), and may produce an impairment of synaptic plasticity and cognitive function correlated to the activity of the hippocampal formation (Kogan et al., 2000; Schrijver and Würbel, 2001; Frisone et al., 2002; Bartesaghi, 2004; Talani et al., 2011). In our previous studies on C57BL/6J mice, we showed that SI was associated with a reduced membrane excitability and NMDA receptor-dependent long-term potentiation (LTP) in CA1 pyramidal neurons (Sanna et al., 2011; Talani et al., 2011). These effects were prevented by a prolonged treatment with the neuroactive steroid precursor progesterone, suggesting that the reduced levels of these hormones in isolated animals could play an important role in the regulation of hippocampal circuit activity (Talani et al., 2011).

Considering the well-known tri-synaptic circuitry of the hippocampal formation (DG-CA3-CA1; Amaral and Witter, 1989), in the present work, we sought to further investigate electrophysiologically the effects of post-weaning SI in C57BL/6J mice on the intrinsic membrane properties and excitability of DG granule cells, together with an evaluation of both GABAergic inhibitory and glutamatergic excitatory inputs on these cells. The data obtained in this work, jointly with those previously published (Sanna et al., 2011; Talani et al., 2011), led us to suggest that the reduced levels of neuroactive steroids in the hippocampus of isolated mice promote a potentiation of GABAergic tonic currents in DG granule cells which, together with a reduced glutamatergic inputs, reduces the excitability level of these cells. The latter effect, in turn, may contribute to hamper both excitability and LTP formation in pyramidal neurons of the CA1 field (Sanna et al., 2011; Talani et al., 2011).

## Materials and Methods

### Animals

C57BL/6J mice (Charles River, Como, Italy) were bred in our animal facility and maintained under an artificial 12-h-light, 12-h-dark cycle (lights on from 08:00 to 20:00 h), at constant temperature of 22° ± 2°C, and a relative humidity of 65%. They had free access to water and standard laboratory food at all time. Animal care and handling throughout the experimental procedures were in accordance with the European Communities Council Directive of 24 November 1986 (86/609/EEC). The experimental protocols were also approved by the Animal Ethics Committee of the University of Cagliari.

### Protocol of Isolation Rearing

Newborn mouse pups were left undisturbed with their mothers until weaning (PND21). Male mice were then randomly assigned to be housed 4–6 per cage (group-housed, GH), or one per cage (socially isolated, SI) for 6 weeks. In a separate set of experiments, GH and SI mice were injected subcutaneously once a day with progesterone (5 mg/kg, dissolved in 20% β-cyclodextrin) throughout the entire 6-week period of isolation; control SI mice received an equivalent injection of the vehicle solution according to the same schedule. Only for the measurement of hormone levels, a set of GH and SI mice treated with progesterone were also co-administered with finasteride (30 mg/kg, s.c.).

### Measurement of Hormone Levels

Mice (5 for each experimental group) were sacrificed at 03:00 p.m., 6 h after the last drug injection, with the same procedure used for the brain slice preparation (see below). Blood samples were collected from the trunk of killed mice into K3-EDTA tubes, centrifuged at 900× g for 10 min at 4°C and frozen at −80°C until use. Levels of progesterone and 3α,5α-THP were assayed with the competitive enzyme immunoassay in the collected plasma. ELISA was performed according to the manufacturer’s instructions for progesterone (Progesterone rat/mouse ELISA, Demeditec, Germany) and 3α,5α-THP (Enzyme-linked Immunosorbent Assay Kit for 3α,5α-THP, Cloud-Clone corp, USA) using a 96-well plate that was precoated with a polyclonal anti-progesterone antibody and monoclonal antibody specific to 3α,5α-THP, respectively.

Progesterone ELISA: the kit provided a six-point standard curve using two-fold serial dilutions. Each sample was run in duplicate. An unknown amount of progesterone present in the sample and a defined amount of progesterone conjugated to horseradish peroxidase (HRP) compete for the binding sites of progesterone antiserum coated to the wells of a microplate. After incubation on a shaker samples and standard point wells were washed four times. After addition of the substrate solution the concentration of progesterone is calculated with a 4-parameter logistic curve.

3α,5α-THP ELISA: the kit provided a six-point standard curve using two-fold serial dilutions. Each sample was run in duplicate. A competitive inhibition reaction is started between biotin labeled 3α,5α-THP and unlabeled 3α,5α-THP (standards or samples) with the pre-coated antibody specific to 3α,5α-THP. After 1 h incubation at 37°C the unbound conjugate is washed off. Subsequently, avidin conjugated to HRP was added to samples and standard point wells and incubated 30 min at 37°C. The amount of bound HRP conjugate is inversely proportional to the concentration of 3α,5α-THP in the sample. After addition of the substrate solution, the intensity of color developed is inversely proportional to the concentration of 3α,5α-THP in the samples.

The enzymatic colorimetric reaction was assessed at 400 nm through a Victor X5 plate reader (Perkin-Elmer), and resulting values were extrapolated by a 4-parameter logistic curve.

### Preparation of Mouse Hippocampal Slices

Hippocampal slices were prepared from GH and SI mice as previously described (Sanna et al., 2011). In brief, animals were subjected to deep anesthesia with chloroform and decapitated. Their brain was rapidly removed from the skull and transferred to a modified artificial cerebrospinal fluid (ACSF) containing (in mM): 220 sucrose, 2 KCl, 0.2 CaCl_2_, 6 MgSO_4_, 26 NaHCO_3_, 1.3 NaH_2_PO_4_, and 10 D-glucose (pH 7.4, set by aeration with 95% O_2_ and 5% CO_2_). Coronal brain slices (thickness, 300 μm) containing the dorsal hippocampus were cut in ice-cold modified ACSF with the use of a Leica VT1200S vibratome (Leica, Heidelberg, Germany). The slices were then transferred immediately to a nylon net submerged in standard ACSF for at least 40 min at a controlled temperature of 35°C. After subsequent incubation for at least 1 h at room temperature, hemi-slices were transferred to the recording chamber, and continuously perfused with standard ACSF at a constant flow rate of ~2 ml/min. For all recordings, the temperature of the bath was maintained at 33°C.

### Whole-Cell Patch-Clamp Recordings

Whole-cell patch-clamp recordings from DG granule cells were performed as previously described (Sanna et al., 2011). Recording pipettes were prepared from borosilicate capillaries with an internal filament with the use of a Fleming Brown micropipette puller (Molecular Devices, Novato, CA, USA). Resistance of the pipettes ranged from 4.5 to 6.0 MΩ when they were filled with either of the two following internal solutions: for current-clamp experiments we used an internal solution containing 135 mM potassium gluconate, 10 mM MgCl_2_, 0.1 mM CaCl_2_, 1 mM EGTA, 10 mM Hepes-KOH (pH 7.3), and 2 mM ATP (disodium salt); for voltage-clamp experiments we used an internal solution containing, in mM, 140 CsCl, 2 MgCl, 2 CaCl, 10 EGTA, 10 HEPES, 2 ATP-Na, pH 7.3 with CsOH 5 N. Only recordings with access resistance of <25 MΩ (values usually ranged from 9 to 20 MΩ) were analyzed. Series resistance was not compensated, and cells were excluded from a further analysis if access resistance changed by >20% during the course of the recording. Recording of the different neurophysiological parameters usually started at least 10 min after the whole cell configuration (membrane patch break-in) was reached. Membrane potentials and membrane currents were recorded with the use of an Axopatch 200-B amplifier (Axon Instruments), filtered at 2 kHz, and digitized at 5 kHz. The pClamp 9.2 software (Molecular Devices, Union City, CA, USA) was used, which allowed us to measure and analyze various kinetic parameters of the neuronal membrane, membrane potentials and currents. For current-clamp experiments, we applied a protocol consisting of the injection of currents of 400-ms duration and ranging in intensity from −80 to 160 pA, with steps of 20 pA, in order to hyperpolarize or depolarize the cell membrane and thus measure voltage changes. Bridge balance compensation as well as pipette capacitance neutralization were applied for these recordings. The parameters analyzed included resting membrane potential, membrane time constant, membrane capacitance, action potential (AP) threshold (defined as the corresponding V value for a dV/dt value of 5 V/s), minimum injected current capable of evoking the first AP, AP latency (time required for the first AP to occur in response to depolarization) and AP frequency. In addition, APs were analyzed for their amplitude, duration and after-hyperpolarization phase (AHP), where the membrane potential falls below the normal resting potential. In addition, membrane input resistance (*R*_in_) was measured in voltage-clamp mode, through the analysis of *I/V* relation; hyperpolarizing voltage steps, ranging from −65 to −85 mV were imposed and steady-state currents required for holding the membrane potential were measured. *R*_in_ was calculated as the slope^−1^ of the linear regression. In order to record hyperpolarization activated cyclic nucleotide-gated (HCN)-mediated *I*_h_, more hyperpolarized voltage steps (up to −105 mV) were applied. For the experiments evaluating changes in GABAergic tonic current in DG granule cells from the different animal groups, after initiation of the whole-cell recording, 10 min were allowed in order for the response to stabilize, and then, recorded baseline activity for approximately 3 min followed by the bath perfusion for 6 min of the GABA_A_R partial agonist α-(4,5,6,7-tetrahydroisoxazolo[5,4-c] pyridin-3-ol; THIP; 3 μM) to increase GABAergic tonic currents. After THIP perfusion, the GABA_A_R antagonist bicuculline (20 μM) was added to block both phasic and tonic currents. As for GABA-mediated currents, also spontaneous and evoked excitatory postsynaptic currents (sEPSC and eEPSC, respectively) were evaluated. The stimulating bipolar concentric electrode was placed in the molecular layer approximately at 400 μm from the neuron under test in order to stimulate mainly the glutamatergic afferents belonging to the perforant pathway. Paired-pulse (PP) protocol was also used with an interstimulus interval of 50 ms, and this paired stimulation was repeated at 20 s intervals. The amplitude ratio of the second to the first eEPSC evoked by PP stimulation was defined as the PP ratio and was calculated on the average of three consecutive sweeps.

### Statistical Analysis

Data are presented as Mean ± standard error of the mean (SEM) and were compared by one-way or two-way analysis of variance (ANOVA) followed by Bonferroni’s *post hoc* test, or Student’s *t* test with the use of Prism software (version 6, Graphpad). A *p* value of <0.05 was considered statistically significant.

## Results

### Plasma Levels of Progesterone and 3α,5α-THP and Effect of Treatment with Progesterone and Finasteride

**Figure 1:**
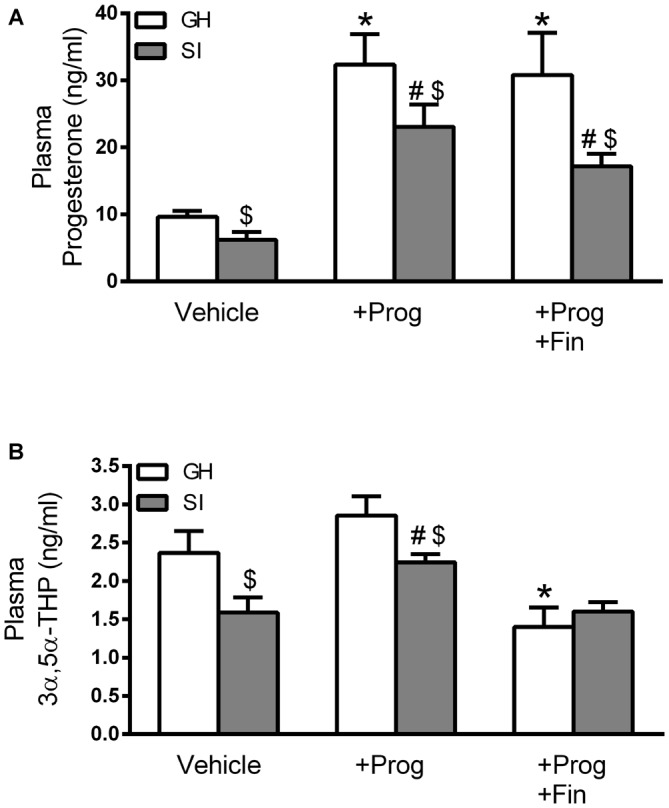
**Changes in plasma progesterone and 3α,5α-THP concentrations induced by social isolation (SI) and effect of progesterone treatment. (A)** Bar graph representing the changes in progesterone concentration measured in the plasma of mice from the different experimental groups (*n* = 5 mice per group). **(B)** Bar graph representing the change in 3α,5α-THP concentration measured in the plasma of the different experimental groups (*n*, 5 animals per group). Data are reported as means ± standard error of the mean (SEM). *p < 0.05 vs. group-housed (GH), ^#^*p* < 0.05 vs. SI (one-way analysis of variance (ANOVA), and Bonferroni’s *post hoc* test), ^$^*p* < 0.05 vs. GH similarly treated (*t*-test).

Rearing C57BL/6J mice in isolation for 6 weeks, starting at the time of weaning (PND 21), has been consistently shown to produce a sustained reduction in plasma and brain concentrations of GABAergic neuroactive steroids such as 3α,5α-THP and THDOC in rats (Serra et al., 2000, 2005, 2006) and mice (Matsumoto et al., 1999; Agís-Balboa et al., 2007; Sanna et al., 2011). In the present study, we further confirmed these results by measuring a significant (*p* < 0.05) reduction in the plasma concentrations of both progesterone (36%) and 3α,5α-THP (33%) in SI compared to GH control mice (Figure 1). Prolonged treatment with progesterone (5 mg/kg, s.c.) during the isolation exposure resulted in a marked elevation of its plasma concentration in both GH (one-way ANOVA, *F*_(2.9)_ = 10.14; *p* < 0.01) and SI (*F*_(2,8)_ = 16.98; *p* < 0.001) animals which was not significantly altered by the co-treatment of finasteride (30 mg/kg, s.c.), an inhibitor of 5α-reductase, the enzyme involved in the conversion of progesterone in 3α,5α-THP (Russell and Wilson, 1994; Figure 1A). Treatment with progesterone, while modestly affecting plasma 3α,5α-THP levels in GH mice, caused a significant (*p* < 0.05) elevation of this neurosteroid in SI mice reaching an average concentration similar to that detected in the plasma of vehicle-treated GH animals (Figure 1B). Co-administration of progesterone and finasteride resulted, when tested in GH mice, in plasma 3α,5α-THP concentrations falling below of those found in vehicle-treated animals and, in SI mice, in values similar to those observed in vehicle-treated SI animals (Figure 1B).

### SI Increases GABAergic Tonic Currents in DG Granule Cells: Reversal by Progesterone Treatment

**Figure 2:**
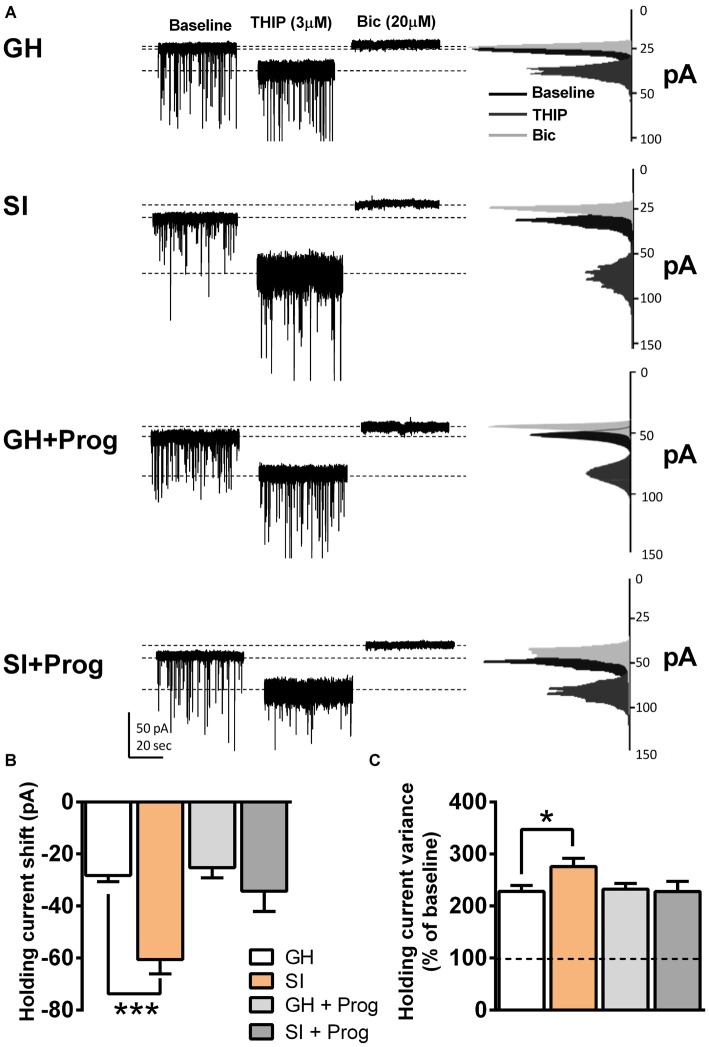
**Effects of SI on GABAergic tonic currents in dentate gyrus (DG) granule cells: reversal by progesterone treatment. (A)** Representative traces of GABAergic currents in the whole-cell voltage-clamp mode (holding potential, −65 mV) from DG granule cells present in slices obtained from mice of the different experimental groups. After a baseline of 3 min, bath perfusion of THIP (3 μM) induced an increase in holding current variance and a negative shift in the holding current. All GABAergic currents were blocked by 20 μM bicuculline (Bic). On the right, the current distribution graphs relative to each of the three different conditions (baseline, THIP and Bic) are shown. **(B,C)** Bar graphs summarizing the changes in holding current **(B)** and holding current variance **(C)**. Data are presented as Mean ± SEM [*n*, 15 (GH), 26 (SI), 7 (GH+Prog), 7 (SI+Prog)] . *p < 0.05, ***p < 0.001 vs. GH (one-way ANOVA, and Bonferroni’s *post hoc* test).

Consistent with previous observations obtained in our laboratory in both rats (Serra et al., 2006) and mice (Sanna et al., 2011), we found that SI induced a significant enhancement of the stimulatory effect of the GABA_A_R agonist THIP (3 μM) on GABAergic tonic currents recorded in DG granule cells, compared to the effect observed in the same neurons obtained from GH animals (Figures 2A–C). The changes induced by SI on the modulatory action of THIP comprised a larger shift in holding current (*p* < 0.001; Figure 2B) as well as a greater increase in the percentage of holding current variance compared to the relative values at baseline (*p* < 0.05; Figure 2C). Daily treatment of mice with progesterone (5 mg/kg, s.c.), throughout the isolation rearing procedure, while producing no significant alterations in GH animals, completely abolished the changes induced by SI on the stimulatory effects of THIP on holding current shift (*p* = 0.3492) and holding current variance (*p* = 0.9811), with values that were not statistically different from those of GH animals (Figures 2A–C).

### Progesterone Treatment Restores the SI-Induced Alterations of DG Granule Cell Membrane Passive Properties

**Figure 3:**
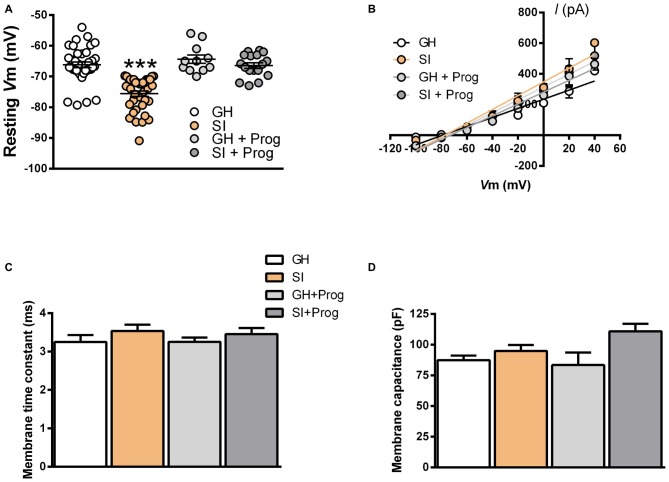
**Effects of SI on intrinsic membrane properties in DG granule cells: reversal by treatment with progesterone. (A)** Scatter plot representing the values of resting membrane potential of DG granule cells from the different experimental groups [*n*, 38 (GH), 37 (SI), 11 (GH+Prog), 17 (SI+Prog)], ***p < 0.001 vs. GH (one-way ANOVA, and Bonferroni’s *post hoc* test). **(B)** *I****/****V* curves of evoked GABAergic inhibitory postsynaptic currents (IPSCs) with the *E*_Cl_ indicated by the intercepts with the *V*-axis (*n*, 7 neurons per each group). **(C,D)** Bar graph representing the values of membrane time constant **(C)** and membrane capacitance **(D)** recorded in neurons from the different experimental groups [*n*, 30 (GH), 39 (SI), 29 (GH+Prog), 13 (SI+Prog)].

Our previous study demonstrated that the increase in GABA_A_R-mediated tonic currents detected in DG granule cells of SI mice is associated with an enhanced expression of α4/δ-containing GABA_A_R in these neurons (Sanna et al., 2011). Thus, we subsequently attempted to determine the impact of SI-induced increase in GABA_A_R-mediated tonic currents in DG granule cells on the basal neurophysiological properties of these neurons. Resting membrane voltage (*V*m) measured in DG granule cells from SI mice (−78 ± 1.2 mV) was significantly (*p* < 0.001) altered when compared to that found in cells from GH animals (−68 ± 1.1 mV; Figure 3A). Such hyperpolarized resting *V*m was not associated with a change in the electrochemical gradient of Cl^−^ ions since the *I/V* curve for evoked GABAergic inhibitory postsynaptic currents (IPSCs) revealed that the value of *E*_Cl_ was not different in DG granule cells obtained from GH (−82 mV) and SI mice (−81 mV; Figure 3B). Measurement of membrane time constant and membrane capacitance in DG granule cells did not reveal significant alterations in SI compared to GH mice (Figures 3C,D).

**Figure 4:**
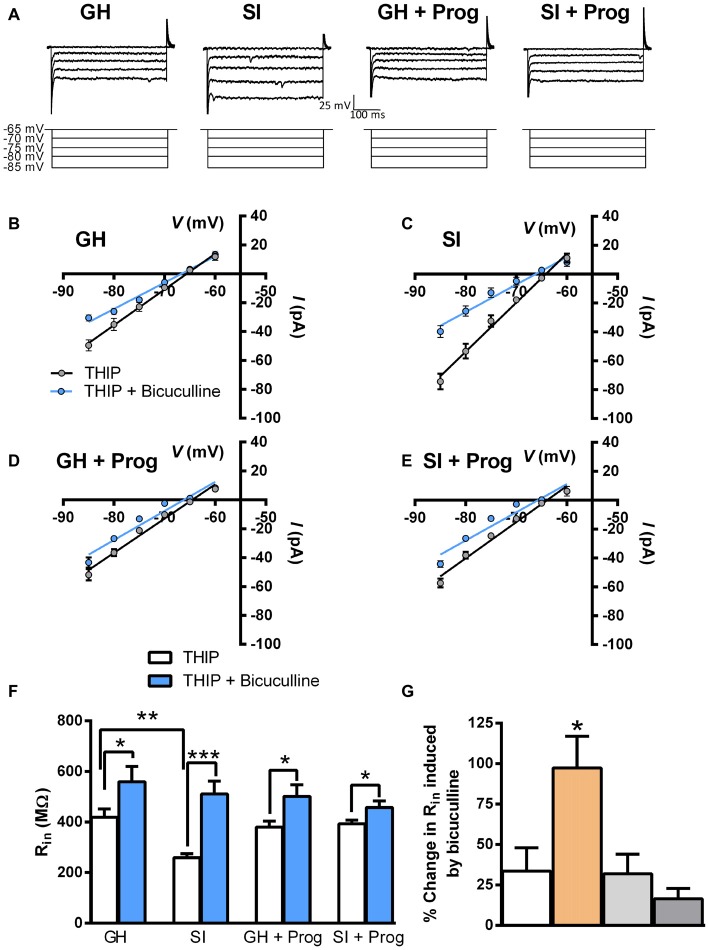
**Effect of SI and progesterone treatment on input resistance in DG granule cells. (A)** Representative traces of holding current changes in response to hyperpolarizing voltage steps (from −65 to −85 mV, Δ 5 mV) in a sample neuron from each experimental group. **(B–E)** Scatter plot of all experimental groups representing the *I/V* curves in the presence of 1 μM THIP without and with the application of 20 μM bicuculline. **(F)** Bar graph of the averaged *R*_in_ from the *I/V* curves graphed in **(B–E)** [*n*, 16 (GH), 14 (SI), 11 (GH+Prog), 10 (SI+Prog)]. **(G)** Bar graph of the averaged effect of bicuculline on *R*_in_ in all experimental groups (*n*, 5 neurons per each group). *p < 0.05; **p < 0.001; ***p < 0.0001 vs. GH (one-way ANOVA, and Bonferroni’s *post hoc* test).

We then reasoned that, in DG granule cells of SI mice, an increase of inhibitory tonic currents, likely consequent to an enhanced surface expression of extrasynaptic α4/δ-containing GABA_A_R (Sanna et al., 2011), which, when stimulated in response to binding of ambient concentrations of GABA, should be continuously activated and the associated Cl^−^ channels be tonically in the open state (see Belelli et al., 2009), could result in an alteration of membrane properties such as decreased membrane input resistance (*R*_in_). Because of the different resting membrane potential between DG granule cells from GH and SI mice, *R*_in_ was measured in the voltage-clamp mode by which the somatic *V*m is controlled and allows for a precise *I/V* curve (Figure 4A). Recordings were performed first in the presence of 1 μM THIP, in order to strengthen preferentially the activity of α4/δ-containing GABA_A_R, and subsequently in the presence of both THIP and 20 μM bicuculline to block the activity of all GABA_A_R. The *I/V* curves, both in the presence of THIP and THIP plus bicuculline, performed in DG granule cells from mice of the different experimental groups, are shown in Figures 4B–E, where the relative *R*_in_ value is calculated as the slope^−1^ of the linear regression. The summary graph in Figure 4F shows that SI significantly (*p* < 0.01) reduced *R*_in_ (259 ± 16 MΩ) compared to the value (419 ± 32 MΩ) found in GH mice. As expected, bicuculline significantly (*p* < 0.05) increased *R*_in_ in all cells tested, but this effect resulted much larger (*p* < 0.001) in those from SI mice (Figure 4G). Bath-perfusion of bicuculline also abolished the difference (*p* = 0.55) in *R*_in_ between GH (560 ± 60 MΩ) and SI (511 ± 51 MΩ; Figures 4F,G). Interestingly, the daily treatment of mice with progesterone, while being ineffective in GH mice, caused the reversal of the changes in DG granule cell resting membrane voltage (67 ± 1.7 mV) and *R*_in_ (393 ± 15 MΩ) induced by SI (Figures 3A, 4).

### Effects of SI on *I*_h_ Recorded in DG Granule Cells

**Figure 5:**
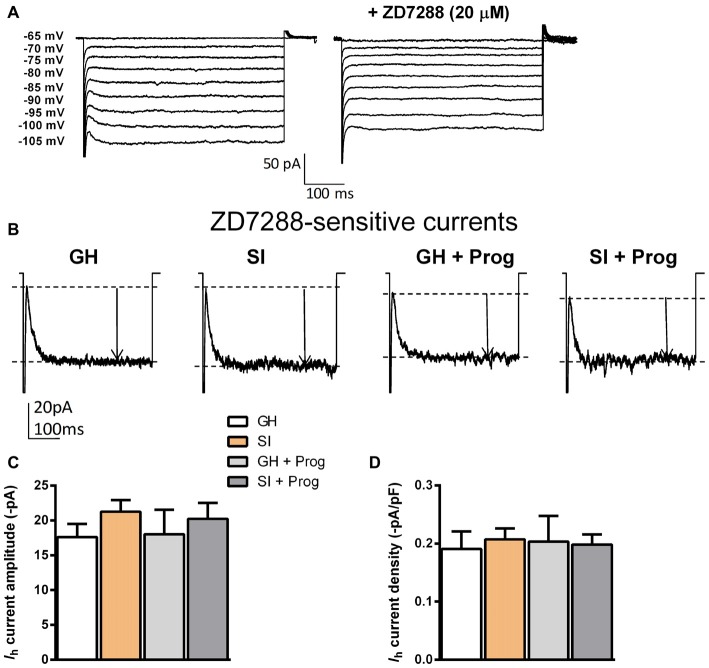
**Effect of SI and progesterone treatment on *I*_h_ recorded in DG granule cells. (A)** Representative traces obtained from a single DG granule cell from GH mice in the absence (left) and presence (right) of the hyperpolarization activated cyclic nucleotide-gated (HCN) selective antagonist ZD7288 (20 μM). **(B)** Representative traces of *I*_h_ recored at −105 mV. Current amplitude is highlighted by the black arrow. **(C,D)** Bar graph representing the change in *I*_h_ amplitude in absolute values (−pA) **(C)** and as current density (pA/pF) **(D)** in the different neurons in all experimental groups [*n*, 16 (GH), 21 (SI), 10 (GH+Prog), 10 (SI+Prog)].

While performing preliminary voltage-clamp recordings in DG granule cells from GH mice, we realized that these cells show *I*_h_ of a small amplitude mediated by HCN channels at membrane potentials more hyperpolarized than −85 mV (Figure 5A). These currents were in fact identified as *I*_h_ because bath perfusion of the selective HCN inhibitor ZD7288 (20 μM) caused their suppression. To test whether differences in *I*_h_ could potentially account for the altered *R*_in_ found in SI mice, such currents were measured in cells from the different experimental groups. By taking into account both absolute amplitude and current density (current amplitude/membrane capacitance) of *I*_h_ generated at a potential of −105 mV, the results showed no significant difference between granule cells from the various experimental groups (Figures 5B–D).

### Effects of SI on DG Granule Cell Excitability

**Figure 6:**
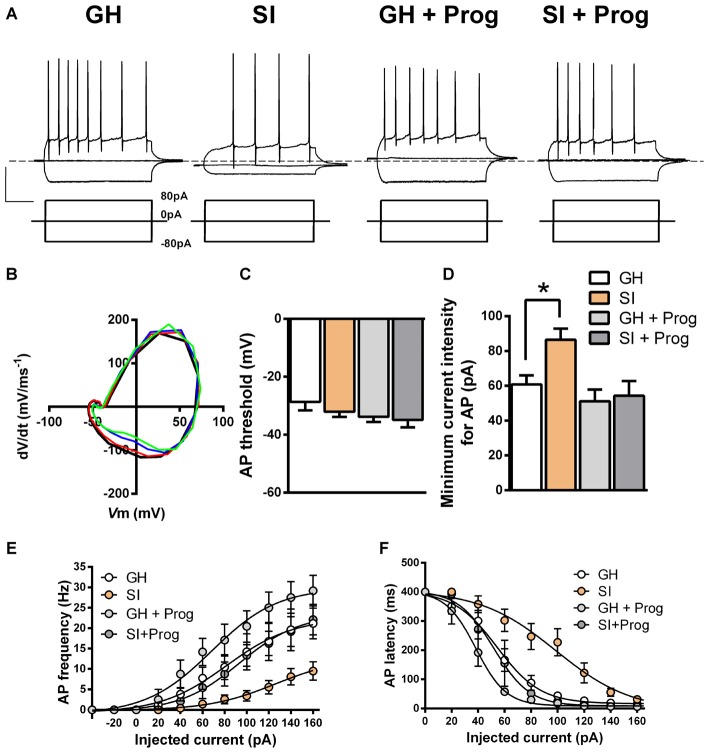
**Effects of SI on DG granule cell excitability: reversal by progesterone treatment. (A)** Representative membrane voltage responses to negative (−80 pA) and positive (80 pA) current pulses applied to DG granule cells from different experimental groups. **(B)** Phase plots representing the change in dV/dt at different values of *V*_m_ in a representative cell from each experimental group (GH black, SI red, GH + Prog blue, and SI + Prog green). **(C)** Bar graph of action potential (AP) threshold calculated as *V*_m_ at which dV/dt has a value of 5 V/s. **(D)** Minimum current intensity required for induction of the first AP **(E)**. AP frequency and **(F)** AP latency. Data are means ± SEM [*n*, 24 (GH), 28 (SI), 9 (GH + Prog), 7 (SI + Prog)]. *p < 0.05 vs. GH (one-way ANOVA, and Bonferroni’s *post hoc* test).

**Figure 7:**
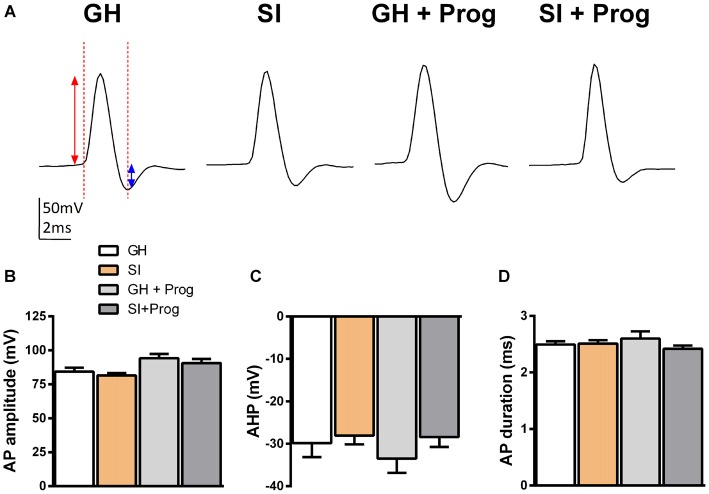
**Lack of effects of SI and progesterone treatment on AP parameters. (A)** Representative traces of an evoked AP in response to an over-threshold depolarizing voltage step. The red arrow indicates the AP amplitude, the blue arrow indicates the amplitude of the after-hyperpolarization potential (AHP), while the vertical red line indicates the AP duration. **(B–D)** Bar graphs representing the averaged values of AP amplitude **(B)**, AHP **(C)** and AP duration **(D)** obtained from DG granule cells of the different experimental groups [*n*, 32 (GH), 49 (SI), 10 (GH+Prog), 17 (SI+Prog)].

The findings that DG granule cells are hyperpolarized and their *R*_in_ is reduced in mice exposed to SI, when compared to GH animals, suggest that these alterations in the membrane properties may be important also for modulating neuronal excitability and, in turn, neuronal firing. In order to investigate this hypothesis, additional current-clamp experiments were performed in DG granule cells with the injection of depolarizing currents of increasing intensity (Figure 6A) starting at the actual *V*m to include any basal changes of membrane potential. Despite AP threshold did not differ significantly between SI (−32 ± 1.8 mV; *n* = 25) and GH (−29 ± 2.8 mV; *n* = 18) mice (Figures 6B,C), we found a significant (*p* < 0.05) increase in the intensity of the injected current needed to fire the first AP in DG granule cells from SI mice (86 ± 6.4 pA) compared to the value found in GH animals (61 ± 5.2 pA; Figure 6D). Furthermore, in the same DG granule cells from SI mice there was a significant decrease (*F*_(3,41)_ = 3.69, two-way ANOVA, *p* < 0.05) in AP frequency and an enhanced (*F*_(3,44)_ = 9.76, two-way ANOVA, *p* < 0.001) AP latency, the latter measured as the time delay (ms) from current injection to the appearance of the first AP (Figures 6A,E,F). Again, treatment with progesterone, while producing no significant effects in GH mice, reverted the changes in DG granule cell excitability induced by SI (Figures 6A–F). Moreover, progesterone treatment caused a modest reduction in *V*m threshold in SI mice, but this effect was not significantly (*p* = 0.118) different from GH animals. A more detailed kinetic analysis of APs generated in response to injection of positive currents, revealed no significant variations among DG granule cells obtained from the different experimental groups in AP amplitude, AHP amplitude, and AP duration (Figure 7).

### Effects of SI on Glutamatergic Transmission in DG Granule Cells

**Figure 8:**
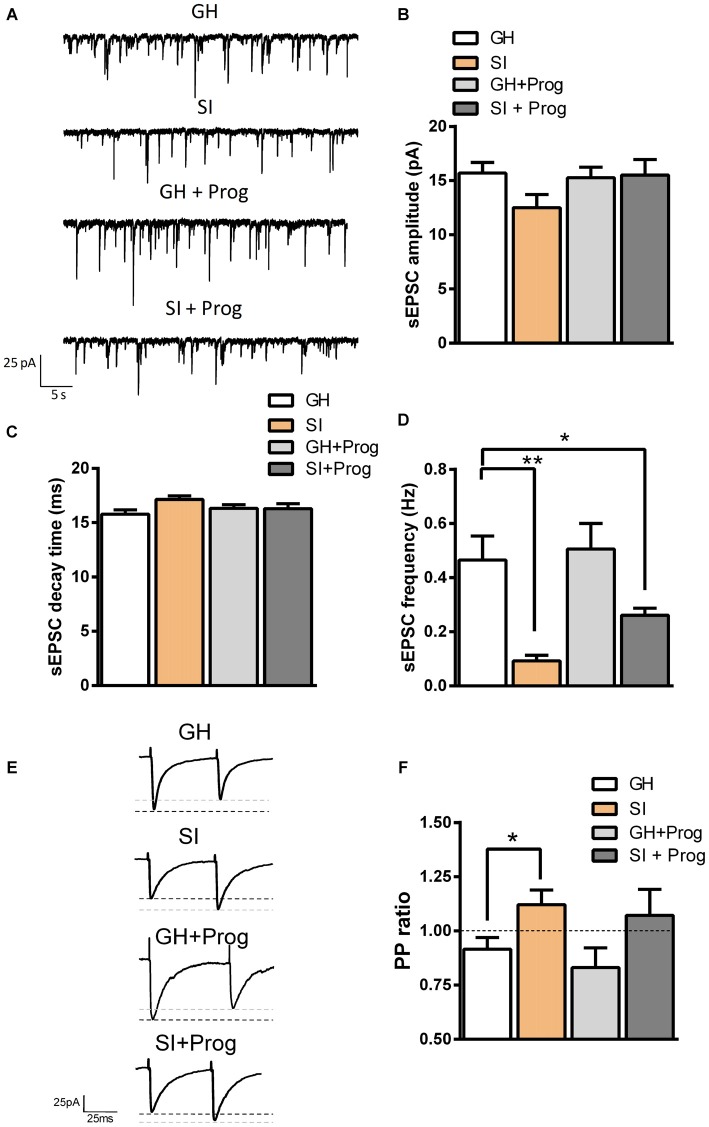
**SI alters glutamatergic transmission in DG granule cells: effect of progesterone treatment. (A)** Representative traces of spontaneous excitatory postsynaptic currents (sEPSCs) recorded in voltage-clamped (−65 mV) DG granule cells of the different experimental groups. **(B–D)** Bar graphs summarizing the effects of SI and progesterone treatment on sEPSC amplitude **(B)**, decay time constant **(C)**, and frequency **(D)**. Data are expressed as absolute values and are Mean ± SEM [*n*, 33 (GH), 22 (SI), 25 (GH+Prog), 19 (SI+Prog)]. **(E)** Representative traces of glutamatergic EPSCs evoked by paired-pulse (PP) protocol with an inter-interval of 50 ms. **(F)** Bar graph summarizing the averaged PP ratio of evoked EPSCs (eEPSCs) in DG granule cells from the different experimental groups. Data are Mean ± SEM [*n*, 15 (GH), 17 (SI), 6 (GH+Prog), 6 (SI+Prog)]. *p < 0.05; **p < 0.01 vs. GH, one-way ANOVA and Bonferroni’s *post hoc* test.

We next recorded in voltage-clamped (−65 mV) DG granule cells from mice belonging to the different experimental groups, spontaneous glutamatergic EPSCs (sEPSCs), which were pharmacologically isolated by the presence of the GABA_A_R antagonist bicuculline (20 μM; Figure 4A). Analysis of the kinetic properties revealed that there was no significant difference in the amplitude or decay-time of sEPSCs between DG granule cells obtained from GH and SI mice (Figures 8A–C). However, we found a significant (*p* < 0.01) decrease (80%) in sESPC frequency in DG granule cells from SI mice compared to GH animals (Figures 8A,D). Interestingly, the difference between GH and SI mice, although reduced (44%), remained statistically different after progesterone treatment (*p* < 0.05; Figures 8A–D).

Because the reduction in sEPSC frequency found in SI mice may be indicative of an altered probability of glutamate release from presynaptic terminals impinging on DG granule cells, we next recorded electrically evoked EPSCs in DG granule cells and applied the (PP) protocol. Two stimuli with the same intensity, and with an inter-interval of 50 ms, were delivered to afferent fibers, mostly arising from the perforant path, around 400 μm apart from the recorded neuron, and evoked glutamatergic EPSCs mediated by AMPA/kainate receptors were recorded in voltage-clamped (−65 mV) DG granule cells in the presence of bicuculline (20 μM; Figure 8E). In neurons from GH mice, the PP protocol resulted in a decreased amplitude of the second response, with a PP ratio <1 (PP depression, PPD; Figures 8, 4E,F). Vice versa, in granule cells from SI mice the PP ratio was significantly (*p* < 0.05 vs. GH) higher resulting in a PP facilitation (PPF; Figures 8E,F), an effect consistent with a reduced probability of glutamate release. Similar to what observed in the sEPSC experiments, treatment of SI mice with progesterone failed to change significantly the PP ratio found in vehicle-treated SI mice, but the difference between GH and progesterone-treated SI animals was not statistically significant (Figures 8E,F).

## Discussion

The results of the present work demonstrate that post-weaning SI of C57BL/6J mice is accompanied by marked alterations in the passive membrane properties and excitability of DG granule cells which appear related to the increased GABAergic inhibitory tonic currents. This latter effect, in turn, may result from the sustained decrease in plasma (present work) and hippocampal (Sanna et al., 2011) concentrations of neuroactive steroids that are associated with SI. In fact, progesterone treatment of SI mice, by re-establishing the plasma concentration of 3α,5α-THP to a level similar to that measured in GH animals, was effective in reverting all of these effects. In addition, SI decreased also the frequency of glutamatergic sEPSCs recorded in DG granule cells with a parallel increase in the PP ratio. These data, altogether, are consistent with a reduced probability of glutamate release from presynaptic afferents impinging on DG granule cells. However, these effect were only modestly influenced by progesterone treatment suggesting that regulation of glutamatergic synapses in SI animals does not appear completely related to changes in neuroactive steroid levels.

The finding that the plasma concentration of both progesterone and 3α,5α-THP was decreased in SI C57BL/6J mice is consistent with previous studies showing similar effects in the hippocampus, cerebral cortex, and plasma of both mice (Matsumoto et al., 1999; Agís-Balboa et al., 2007; Sanna et al., 2011), and rats (Serra et al., 2000, 2005, 2006). Treatment of SI mice with progesterone during the SI exposure could restore the plasma concentrations of 3α,5α-THP to values similar to those found in GH control animals. In fact, this was prevented by the co-administration of finasteride which, by inhibiting the 5α-reductase (Russell and Wilson, 1994), blocks the first step in the conversion of progesterone in 3α,5α-THP. Thus, this represents a useful model for testing the role of the decreased neurosteroid levels in the regulation of both GABAergic and glutamatergic synaptic signaling in the DG granule cells and, ultimately, on neuronal excitability in the hippocampal circuit.

The increase in the tonic component of the GABAergic transmission, induced by SI, was previously shown to be related to an elevated expression of α4 and δ subunits of the GABA_A_R (Sanna et al., 2011). Because Cl^−^ channels associated with α4/δ-containing receptors are endowed with a very slow inactivation rate and stay constantly in the open state (see Belelli et al., 2009), we predicted that such effect would determine also marked alterations in the membrane properties as well as in the excitability of DG granule cells from SI mice. The significant reduction in resting membrane potential and input resistance are largely consistent with this idea. This is further supported by the observation that bicuculline, by blocking the activity of all GABA_A_R, produced in SI mice a larger increase in input resistance and, in its presence the difference in input resistance between GH and SI animals was abolished. Interestingly, a similar correlation between increase in GABAergic tonic currents and decrease of membrane input resistance was described in the hippocampal CA1 field of mice during puberty (Shen et al., 2007; Smith, 2013). Reduction of membrane input resistance, in turn, represents a relevant neurophysiological alteration as it will determine a lower change in membrane voltage in response to EPSCs such as those that are activated through the release of glutamate and activation of AMPA/kainate receptors.

Firing of APs induced by membrane depolarization was also greatly attenuated in SI mice, indicating that the increase of GABAergic tonic currents may be responsible for the reduction of DG granule cells membrane excitability. In addition, these data are consistent with those of previous studies showing that SI is associated with pronounced changes in hippocampal neuronal membrane excitability (Moyer et al., 1996, 2000; Thompson et al., 1996; Tombaugh et al., 2005; Talani et al., 2011).

The finding that daily treatment with progesterone is effective in reversing the increase in tonic current, decrease in input resistance and AP firing, observed in SI mice, strongly suggests that the reduced plasma (present work) and hippocampal (Sanna et al., 2011) levels of neuroactive steroids may represent a primary event in association with SI that triggers the up-regulation of extrasynaptic α4/δ GABA_A_R (Sanna et al., 2011). This latter effect, in turn, may be responsible for the decreased neuronal excitability of DG granule cells. In addition, daily progesterone treatment did not affect these parameters in GH animals suggesting that exposure to progesterone only restores the levels of 3α,5α-THP to the values similar to those detected in GH. It is important to note that the decreased hippocampal levels of neuroactive steroids, as a consequence of SI, has been shown to be related to a decreased expression of 5α-reductase, which occurs selectively at the level of the DG and CA3, but not CA1, subregions (Agís-Balboa et al., 2007). Thus, these findings may suggest that SI, by reducing the conversion of progesterone into neuroactive steroids, may regulate locally the expression levels of α4/δ extrasynaptic GABA_A_R in DG granule cells as a compensatory mechanism. Altogether, these results point to the DG as a primary target for the changes produced by SI.

Because the DG is upstream of the hippocampal circuitry, functional alterations at this level may have a profound impact on the activity in other subfields downstream of the hippocampal formation. Preliminary current-clamp experiments performed in our lab indicate that neuronal excitability in the CA3 subfield, the major target of mossy fibers arising from DG granule cells, is markedly reduced in isolated C57BL/6J mice, compared to GH animals (Licheri et al., unpublished data). Consistent with this observation, early isolation rearing of guinea pigs caused market dendritic atrophy (Bartesaghi and Severi, 2002) and reduced excitability of CA3 pyramidal neurons (Bartesaghi, 2004). In addition, we have previously reported that SI is associated with a reduced excitability and LTP formation in the pyramidal neurons of the CA1 subfield (Talani et al., 2011), an effect that could be reversed by progesterone treatment during SI. These observations are also in agreement with those proposed in a previous study in guinea pig hippocampus (Bartesaghi, 2004). Thus, we hypothesize that changes in excitability and long-term plasticity of glutamatergic synapses in the CA1 subfield might be secondary to the changes occurring in the DG granule cells, given the neuronal organization of the trisynaptic nature of the hippocampal formation (Amaral and Witter, 1989).

Moreover, SI also alters glutamatergic transmission at the level of the DG. This effect consisted mainly in a decreased probability of glutamate release from afferents impinging on DG granule cells. This was indexed by both the decreased frequency of sEPSCs and increase in PP ratio, as compared to GH mice. Given that progesterone treatment only partially reduced the effect of SI on sEPSC frequency but did not modify significantly the change in PP ratio, our data do not provide sufficient evidences to suggest that regulation of glutamatergic synapses in SI animals is dependent on changes in neuroactive steroid levels. The potential interaction between SI-induced regulation of glutamatergic transmission and fluctuations of 3α,5α-THP concentrations deserves a more detailed investigation. Interestingly, in the hippocampal formation of SI rats, immunoreactivity for synaptophysin, a presynaptic marker (Wiedenmann and Franke, 1985), was found reduced selectively in the DG, but not CA1 or CA3 subfields (Varty et al., 1999), an effect qualitatively similar to that produced by either the transection of the perforant pathway, which project from the entorhinal cortex to the DG granule cells (Kirkby and Higgins, 1998), or the ablation of the entorhinal cortex (Masliah et al., 1991). Thus, the decrease in sEPSC frequency found in the DG granule cells might be also consequent to a loss of glutamatergic afferents originated from the entorhinal cortex. In addition, other authors have reported that SI decreases glutamate and glutamine concentrations in the rat hippocampus (Shao et al., 2015). Furthermore, the reduced glutamate release, found in our study, may also be associated to a lower level of NMDAR activation with a consequent decrease of the NMDA-dependent synthesis of GABAergic neurosteroids, including 3α,5α-THP, in hippocampal pyramidal neurons (Tokuda et al., 2011), which may also contribute to the decrease of hippocampal excitability and LTP formation in the CA1 subfield (Talani et al., 2011).

Deprivation of social interactions in rodents leads to severe impairments of cognitive functions directly correlated to the normal activity of the hippocampal formation (Kogan et al., 2000; Schrijver and Würbel, 2001; Frisone et al., 2002; Huang et al., 2002; Bartesaghi, 2004; Talani et al., 2011) that plays a crucial role in learning and memory formation; in fact lesions at different subregions of this brain district such as CA1, DG, and/or entorhinal cortex, cause severe cognition impairments (Olton et al., 1982; Squire, 1992; Jarrard, 1993; Alvarez et al., 1995). Overall, these data, together with the findings obtained in our previous studies (Sanna et al., 2011; Talani et al., 2011), indicate that signals arising from the hippocampal circuitry undergo dramatic deterioration in SI C57BL/6J male mice. Furthermore, these findings suggest that exposure to stress during adolescence might cause an impairment in learning and memory function associated to the marked changes in neuronal excitability at the level of the different subregions of the hippocampal formation, which may depend, at least in part, from the altered levels of neuroactive steroids.

## Author Contributions

GT performed the experiments and wrote the article. FB, VLi and VLo performed the experiments. GB and ES wrote the article.

## Funding

The present work was supported by funds provided from RAS (Regione Autonoma della Sardegna) L.R. 7/2007, 2008, Prot. CRP_63; L.R. 7/2007, 2010, Prot. CRP26052.

## Conflict of Interest Statement

The authors declare that the research was conducted in the absence of any commercial or financial relationships that could be construed as a potential conflict of interest.
